# Derivation and validation of diagnostic models for myocardial fibrosis in duchenne muscular dystrophy: assessed by multi-parameter cardiovascular magnetic resonance

**DOI:** 10.1186/s13023-023-02931-y

**Published:** 2023-12-11

**Authors:** Zi-qi Zhou, Hua-yan Xu, Hang Fu, Ke Xu, Rong Xu, Xiao-tang Cai, Ying-kun Guo

**Affiliations:** 1 Department of Radiology, Key Laboratory of Birth Defects and Related Diseases of Women and Children of Ministry of Education, West China Second University Hospital, Sichuan University, 20# Section 3 South Renmin Road, Chengdu, 610041 China; 2 Department of Rehabilitation, West China Second University Hospital, Sichuan University, 20# Section 3 South Renmin Road, Department of Rehabilitation, Chengdu, China

**Keywords:** Duchenne muscular dystrophy, Cardiovascular magnetic resonance, Late gadolinium enhancement, Native T1, Longitudinal strain

## Abstract

**Background:**

Gadolinium-enhanced cardiovascular magnetic resonance (CMR) is the most widely used approach for diagnosing myocardial fibrosis with late gadolinium enhancement (LGE) in cardiomyopathy associated with Duchenne muscular dystrophy. Given the limitations and safety of gadolinium use, we wanted to develop and evaluate multi-parametric pre-contrast CMR models for the diagnosis of LGE and investigate whether they could be utilised as surrogates for LGE in DMD patients.

**Methods:**

A total of 136 DMD patients were prospectively recruited and separated into LGE − and LGE + groups. In the first subset of patients (derivation cohort), regression models for the diagnosis of LGE were built by logistic regression using pre-contrast sequence parameters. In a validation cohort of other patients, the models’ performances were evaluated.

**Results:**

EF, native T1 and longitudinal strain alone, as well as their combinations form seven models. The model that included EF, native T1 and longitudinal strain had the best diagnostic value, but there was no significant difference in diagnostic accuracy among the other models except EF. In the validation cohort, the diagnosis outcomes of models were moderate consistent with the existence of LGE. The longitudinal strain outperformed the other models in terms of diagnostic value (sensitivity: 83.33%, specificity: 54.55%).

**Conclusions:**

Pre-contrast sequences have a moderate predictive value for LGE. Thus, pre-contrast parameters may be considered only in a specific subset of DMD patients who cannot cooperate for long-time examinations and have contradiction of contrast agent to help predict the presence of LGE.

**Trial registration number (TRN):**

ChiCTR1800018340

**Date of registration:**

20180107

**Supplementary Information:**

The online version contains supplementary material available at 10.1186/s13023-023-02931-y.

## Background

DMD is a fatal X-linked recessive muscular degenerative disorder caused by defective dystrophin synthesis, resulting in fibrosis and fat replacement of muscles throughout the body, including skeletal and cardiac muscles, and affects 1 in about every 3500–5000 boys [1]. Most patients die of cardiopulmonary failure in their second to third decades, but with the development of clinical non-invasive mechanical ventilation technology, the risk of death from respiratory failure has been significantly lowered [2] and cardiomyopathy has become the leading cause of death in DMD patients [3].

Non-invasive cardiovascular imaging is indispensable for the early detection of myocardial abnormalities, which can develop early in DMD patients [4]. Currently, CMR has gradually become the preferred imaging technique for cardiac monitoring in DMD patients [5, 6]. LGE of CMR can identify myocardial abnormalities and assess fibrosis before LV dysfunction occurs [7, 8]. Cardiac function in DMD patients with LGE is poorer and declines more quickly than in those without LGE [9]. Furthermore, the existence of LGE is also associated with a significantly increased risk of unfavourable outcomes in DMD patients, including arrhythmias, hospitalization for heart failure and cardiogenic death [8, 10, 11]. Thus, early aggressive management and monitoring of possible LGE before impending LV dysfunction are critical for improving the prognosis of DMD patients. However, DMD patients with abnormal renal function cannot finish this examination because of the renal excretion of GBCAs, essential for LGE imaging [12]. In a small number of people, GBCAs might cause an allergy, resulting in adverse consequences [13]. In addition, GBCAs are also known to deposit in the brain, bones and other organs; there are still concerns regarding the safety of GBCAs in children [14, 15]. As most DMD patients are children and their conditions are often serious, compared with other patients, they are often unable to cooperate for a long time owing to their young age, dyskinesia, scoliosis and other defects caused by disease. As a result, shortening scanning time is an effective way to enhance patient compliance while also obtaining images in good quality. Considering all the above reasons, it is necessary and clinically significant to explore the value of pre-contrast sequences in detecting fibrosis in DMD patients.

Not only cardiac structure and function parameters but also myocardial strain (PS-L: peak strain-longitudinal; PS-C: peak strain-circumferential; PS-R: peak strain-radial), native T1 and T2 values can be obtained by pre-contrast CMR sequences. Previous studies have also shown that LGE is correlated with strain alterations in a variety of diseases, including DMD [16]. The strain value of patients with LGE decreased to a certain extent compared to those without LGE. Furthermore, native T1 mapping allows the quantification of myocardial fibrosis without the administration of GBCAs. Previous research has demonstrated an increase in the native T1 value in DMD patients, which is related to the presence of myocardial fibrosis and LGE [17, 18]. As a result, our study aimed to explore whether different combinations of non-contrast CMR parameters could be utilised as surrogates for LGE to predict the occurrence of LGE in DMD patients, thereby reducing the need for GBCAs and scanning time.

## Methods

### Study population and design

The study complied with the Declaration of Helsinki and was approved by the Biomedical Research Ethics Committee of our medical centre. This study has been registered in clinical trial. And each participant signed an informed consent form prior to the examination. From July 2018 to January 2021, we prospectively collected imaging and clinical data of DMD patients who could undergo standard gadolinium-enhanced CMR scanning in our medical centre. The diagnosis of DMD was confirmed by gene detection or muscle biopsy according to the guideline [3]. The excluding criteria were as follows: (1) the presence of other known coexisting cardiac abnormalities, including hypertrophic cardiomyopathy, dilated cardiomyopathy, congenital heart disease, coronary heart disease, left ventricular non-compaction, hypertensive heart disease, valvular heart disease or other types of cardiomyopathy and myocarditis; (2) poor image quality and inconsistent heart phases; (3) artefacts in the mapping images and LGE appeared in the same layers and (4) incomplete clinical records. Participants were divided randomly into a derivation and a validation cohort. And according to the results of LGE, patients were divided into LGE + and LGE − groups. As a supplementary note, we divided the patients in the derivation cohort into groups < 10 years old and ≥ 10 years old.

### CMR imaging protocol

Standard gadolinium-enhanced CMR scanning was conducted by using a clinical 3.0-T whole-body scanner (MAGNETOM Skyra; Siemens Medical Solutions), with the participant supine, by using the two-element cardiac phased-array coil. All images were acquired during end-expiration breath-holding, and dynamic changes in the ECG and breathing were monitored using the manufacturer’s standard ECG-triggering device and the breath-hold technique. Balanced steady-state free precession (SSFP) sequence (slice thickness = 8 mm, slice gap = 0 mm, repetition time [TR] = 38.42 ms, echo time [TE] = 1.5 ms, flip angle = 35°, matrix = 128 × 256 pixels; field of view [FOV] = 340 × 290 mm^2^) was executed to assess the LV structure and function parameters and tissue-tracking indices were obtained based on 8–12 continuous cine imaging in the short-axis view from the mitral valve level to the LV apex, as well as the horizontal four-chamber and vertical two-chamber long-axis. Native T1 mapping, T2 mapping and LGE images were acquired at the basal, middle and apical standard short-axis levels of the LV. LGE images were obtained 5–10 min after the intravenous injection of gadolinium (Gadovist, Bayer Healthcare) at a dose of 0.1 mmol/kg body weight and a flow rate of 1.2-2.0 ml/s using a single-shot phase-sensitive inversion recovery (PSIR) sequence (slice thickness = 6 mm, slice gap = 3 mm, TR = 2.55 ms, TE = 1.1 ms, flip angle = 55°, matrix = 128 × 192 pixels; FOV = 340 × 360 mm^2^). Native T1 mapping was performed using the Modified Look-Locker inversion recovery (MOLLI) sequence (scanning pattern 5(3)3, slice thickness = 6 mm, slice gap = 3 mm, TR = 2.71 ms, TE = 1.1 ms, flip angle = 35°, matrix = 128 × 192 pixels, FOV = 280 × 224 mm^2^) in combination with motion correction (MOCO) was used to perform T1 mapping quantification before contrast administration [19]. T2 mapping was performed with SSFP (slice thickness = 6 mm, slice gap = 3 mm, TR = 220.69 ms, TE = 1.1 ms, flip angle = 12°, matrix = 144 × 192 pixels, FOV = 280 × 360 mm^2^) to obtain three single-shot images with different T2-preparation times (0, 25, and 55 ms).

### Image analyses

**Fig. 1:**
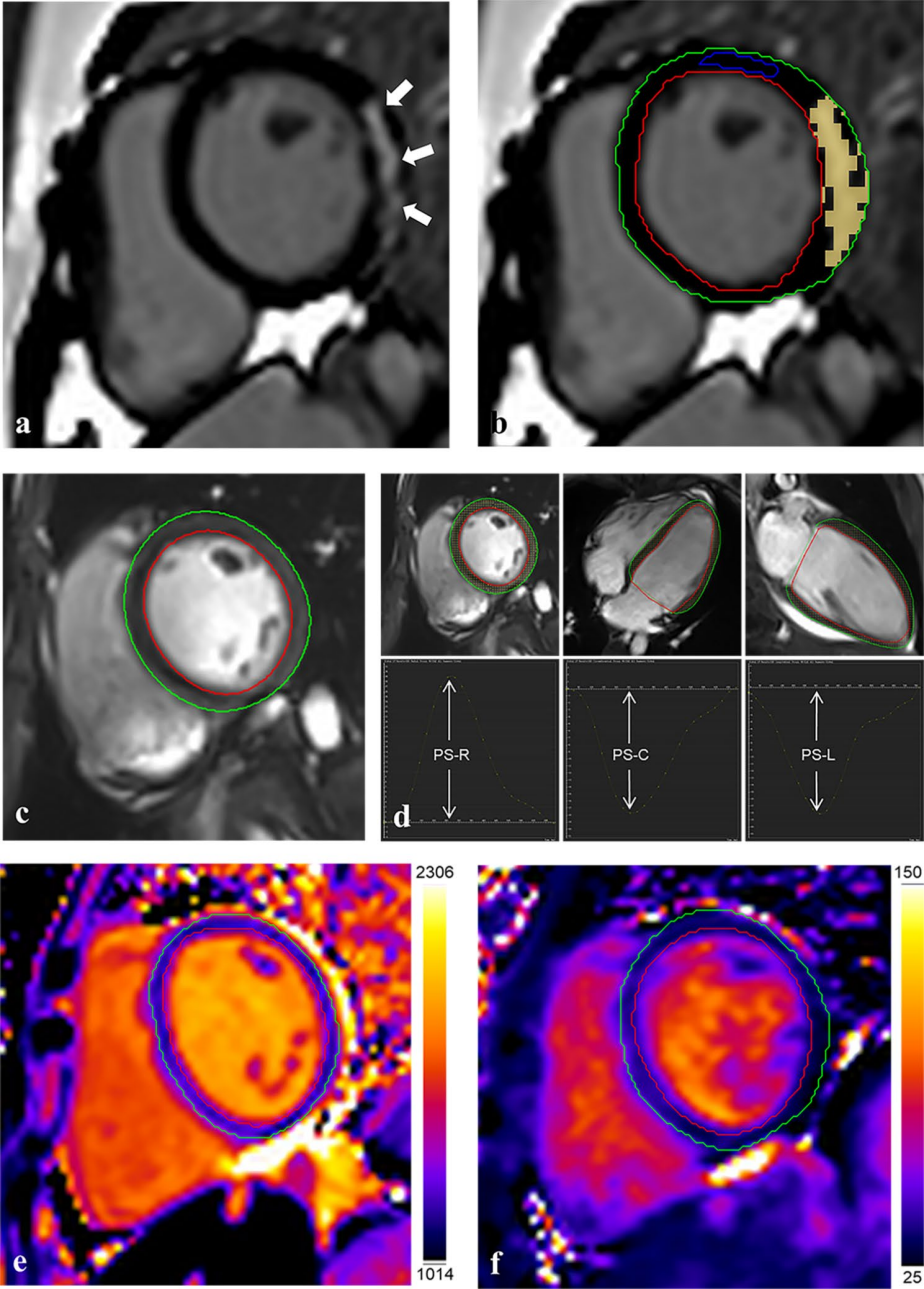
Schematic diagram of partial parameter measurement of CMR Representative CMR-derived parametric images, including LGE in short-axis images (**A**, the white arrows indicate the location of LGE; **B**, to reduce the influence of artifacts, the yellow block represents the LGE range identified by CVI), left ventricular cine images in mid-ventricular slice **(C)**, the peak strain curves in radial, circumferential and longitudinal directions **(D)**, native T1 maps **(E)**, and T2 maps **(F)** in the DMD patient

CMR images were assessed offline using the commercially available post-processing software Cvi42 (Circle Cardiovascular Imaging) and analyzed by an experienced cardiac radiologist with more than 3 years of CMR experience who was blinded to the clinical information. Parameters were calculated automatically by the software or manually according to formulas. In all series, the epi- and endocardial boundaries were delineated manually and papillary muscles were excluded from the myocardium. In order to eliminate the interference of artifacts, LGE was deemed present if myocardial enhancement was confirmed by using a signal intensity threshold of 5SD above the mean signal of the remote normal myocardium through enhanced sequences (Fig. 1-A, B). According to current guidelines [20], short-axis cine images were used to measure cardiac function indices, including LVEF, LV EDV, LV ESV, LV myocardial mass and others (Fig. 1-C). In addition, the LV remodelling index and LV global function index were included for analyses [21]. Short-axis, four-chamber long-axis and two-chamber long-axis cine images were put into the tissue feature tracking module, and the myocardial deformation parameters were measured by automatically tracking the relative motion and displacement of the endocardium and epicardium voxels in the cardiac cycle by delineating the epi- and endocardial boundaries in the diastole, including 2D global radial strain (GRS), 2D global longitudinal strain (GLS) and 2D global circumferential strain (GCS) (Fig. 1-D), which is similar to the principle of using echocardiographic speckle tracking technology to obtain myocardial strain parameters. The end-diastolic phase was the initial position of all strain [22]. Epi- and endocardial boundaries of each slice were delineated on pre-contrast T1 mapping images and T2 mapping images by using the offline T1 and T2 characterization modules respectively (Fig. 1-E, F). Based on the T1 mapping consensus statement, areas of LGE were not excluded [23]. After deleting any layers affected by artefacts, the mean T1 and T2 value of the global myocardium were calculated based on the average of the remaining layers with good image quality.

### Statistical analyses

All statistical analyses were performed using SPSS 22.0 software (International Business Machines) and MedCalc 19.7. Continuous variables were presented as the mean ± standard deviation (SD). Categorical variables were expressed as the frequency and percentage. The normality of the data was determined using Kolmogorov–Smirnov test, and the homogeneity of variance was determined using Levene’s test. Student t-test, Wilcoxon test, and χ2 test were used to compare the variables between groups in baseline and CMR characteristics. All tests were two-sided, and p < 0.05 was considered statistically significant.

*Derivation cohort.* —A subset of patients was used as a derivative cohort to derive models to predict the presence of LGE. Univariate and multivariate logistic regression analyses were performed to calculate the OR and 95% CI. Covariates were incorporated in multivariate logistic regression when p < 0.1 or considered clinically relevant. Any independent factors were combined to calculate a linear regression equation to predict LGE and each regression equation and independent factors were used as a prediction model. ROC curves of different models were constructed to assess appropriate diagnostic thresholds, and the AUC was compared using the Delong method.

*Validation cohort.* —Another subset of patients was used for validation. The threshold value of each model obtained in the derivation cohort was used to determine whether each patient in the validation cohort had LGE, and the results were compared with the imaging diagnostic results. The sensitivity, specificity, negative and positive predictive values of each model in the validation cohort were calculated to evaluate their predictive performance. Kappa test was used to assess the consistency of each model and LGE diagnosis.

## Results

### Demographics and baseline characteristics

A total of 136 patients were enrolled in this study, including 63 LGE + patients. All subjects were male. The derivation cohort contained 96 patients and the remaining 40 DMD boys were included in the validation cohort. In the derivation and validation cohorts, there were 45 and 18 LGE + patients, respectively. In terms of age, height, weight and other baseline characteristics, there were no significant differences between the derivation and validation cohorts (see Additional file 1: Supplementary Table 1).

### Derivation cohort

The baseline characteristics of the patients are shown in Table 1. Compared with LGE − patients, LGE + patients had higher BSA values. Table 2 shows the CMR parameters of patients in derivation cohort. Although the EF of enrolled patients was normal currently (EF > 55%) due to their younger age and mild structural abnormalities of myocardial remodeling, the EF of LGE + patients was significantly lower (58.09 ± 8.81 vs. 62.26 ± 6.25, p = 0.008). Similarly, the EDV, ESV and LV mass of LGE + patients were significantly higher than those of LGE– patients (all p < 0.05). In terms of tissue characterization, global native T1 value significantly increased in LGE + patients (1304.02 ± 62.35 vs.1271.02 ± 46.12, p = 0.001), whereas T2 values did not. Besides, LGE + patients had lower absolute values of PS-R (29.57 ± 4.07 vs. 31.37 ± 4.45, p = 0.043), PS-C (− 18.57 ± 1.95 vs. −19.79 ± 3.07, p = 0.021) and PS-L (− 14.93 ± 2.52 vs. −16.43 ± 2.39, p = 0.004).

**Table 1:** Baseline characteristics of the LGE + (n = 45) and LGE − DMD patients (n = 51)

	DMD LGE + N = 45	DMD LGE− N = 51	P value
***Baseline characteristics***			
**Age, years**	9.16 ± 2.17	8.33 ± 2.30	0.069
**Height, cm**	129.35 ± 11.08	124.69 ± 13.03	0.028*
**Weight, kg**	30.64 ± 8.86	27.26 ± 9.58	0.032*
**BMI, kg/m** ^**2**^	18.09 ± 3.81	17.09 ± 3.33	0.154
**BSA, m** ^**2**^	1.03 ± 0.17	0.90 ± 0.19	0.021*
**Heart rate, bpm**	96.62 ± 14.31	96.50 ± 14.84	0.794
***Medications***			
**Corticosteroids**	34 (75.56)	34 (66.67)	0.339
**ACEI**	9 (20.00)	8 (15.69)	0.581
**β-blocker**	7 (15.56)	10 (19.61)	0.604
**Diuretic**	3 (6.67)	3 (5.88)	0.874

*p＜0.05

Values are presented as mean ± standard deviation or n (%)

**Table 2:** The difference of CMR parameters between LGE + and LGE − DMD patients

	DMD LGE + N = 45	DMD LGE− N = 51	P value
***left ventricle structure and function***			
**SV**	45.11 ± 12.31	43.00 ± 15.80	0.164
**CO**	4.35 ± 1.24	4.02 ± 1.11	0.288
**EF, %**	58.09 ± 8.81	62.26 ± 6.25	0.008**
**EDV, ml**	77.57 ± 17.05	68.65 ± 22.06	0.002**
**ESV, ml**	32.46 ± 9.92	25.65 ± 8.30	＜0.001**
**LV mass, g**	41.76 ± 9.59	38.83 ± 15.53	0.022*
**LV remodeling index**	0.54 ± 0.08	0.57 ± 0.13	0.316
**LVGFI**	47.98 ± 9.63	51.20 ± 7.10	0.043*
***Tissue characterization***			
**Global Native T1, ms**	1304.02 ± 62.35	1271.02 ± 46.12	0.001**
**T2, ms**	36.52 ± 2.05	36.24 ± 3.13	0.602
***Strain***			
**PS-Radial (%)**	29.57 ± 4.07	31.37 ± 4.45	0.043*
**PS-Circumferential (%)**	−18.57 ± 1.95	−19.79 ± 3.07	0.021*
**PS-Longitudinal (%)**	−14.93 ± 2.52	−16.43 ± 2.39	0.004**

*p＜0.05

** p＜0.01

Values are presented as mean ± standard deviation

LVGFI = {LVSV/[(LVEDV + LVESV)/2+(LV mass/1.05)]}*100, LV remodeling index, LV mass/LVEDV; PS, peak strain

Table 3 shows the findings of the univariate and multivariate logistic regression analyses used to confirm the predictors of LGE existence. Although several parameters, such as age, LVGFI, PS-R and PS-C, were univariate predictors of LGE (p < 0.1 in univariable analysis), they were not independent factors after being added to the multivariable regression model. The presence of LGE was only independently correlated with the EF, global native T1 value and PS-L (EF, OR = 0.95, p = 0.05; global native T1 value, OR = 1.01, p = 0.04; PS-L, OR = 0.80, p = 0.02). Moreover, in the group under 10 years old, only longitudinal strain was an independent risk factor for the occurrence of LGE and in addition to longitudinal strain, global native T1 value was also an independent risk factor for LGE in the group over 10 years (see Additional file 1: Supplementary Tables 2 and Table 3).

**Table 3:** Univariable and multivariable logistic regression for the association between LGE and baseline and CMR variables

	Univariable OR (95% CI)	P value	Multivariable OR (95% CI)	P value
**Age**	1.18(0.98–1.42)	0.08§	1.20	0.09
**BMI**	1.08(0.96–1.22)	0.19	1.08	0.31
**HR**	1.00(0.97–1.03)	0.97		
**EF**	0.93(0.87–0.98)	0.01*	0.95(0.89–0.99)	0.05*
**LV remodeling index**	0.15(0.00-6.49)	0.32		
**LVGFI**	0.95(0.91-1.00)	0.07§	1.06	0.31
**Global Native T1**	1.01(1.00-1.02)	0.01**	1.01(1.00-1.02)	0.04*
**T2**	1.04(0.89–1.21)	0.61		
**PS-Radial**	0.90(0.82-1.00)	0.05*	0.94	0.47
**PS-Circumferential**	0.83(0.71–0.98)	0.03*	0.93	0.63
**PS-Longitudinal**	0.78(0.65–0.93)	0.01**	0.80(0.66–0.97)	0.02*

§p＜0.1

*p＜0.05

** p＜0.01

**Fig. 2:**
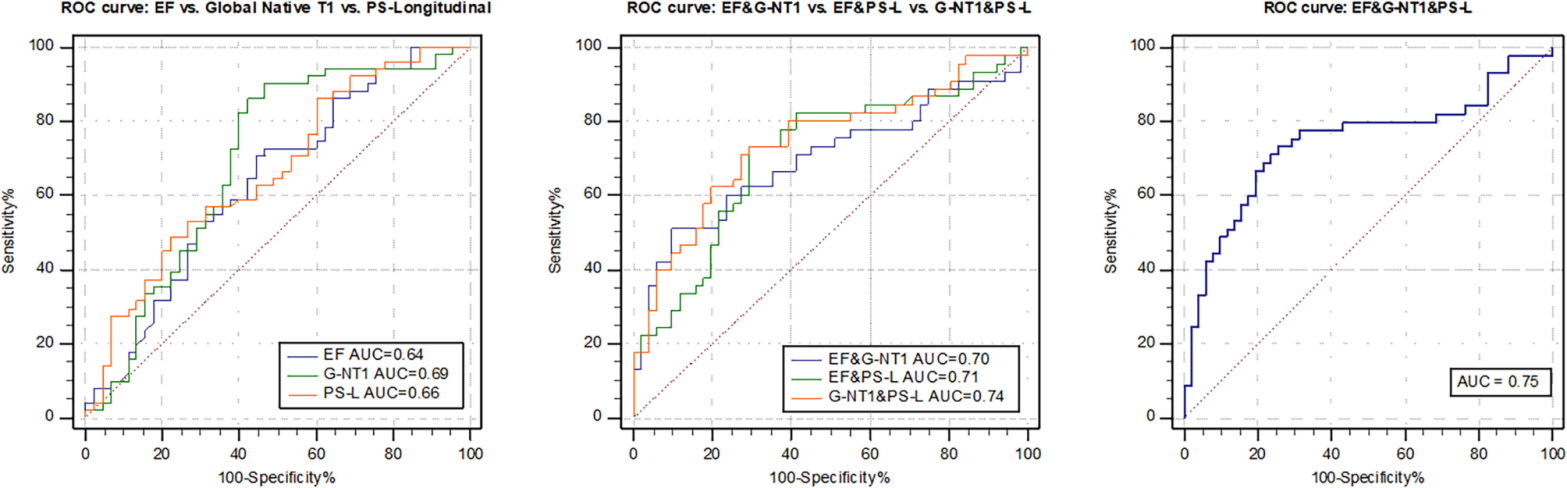
ROC curves for different prediction parameters for differentiating between LGE + and LGE − patients

**Fig. 3:**
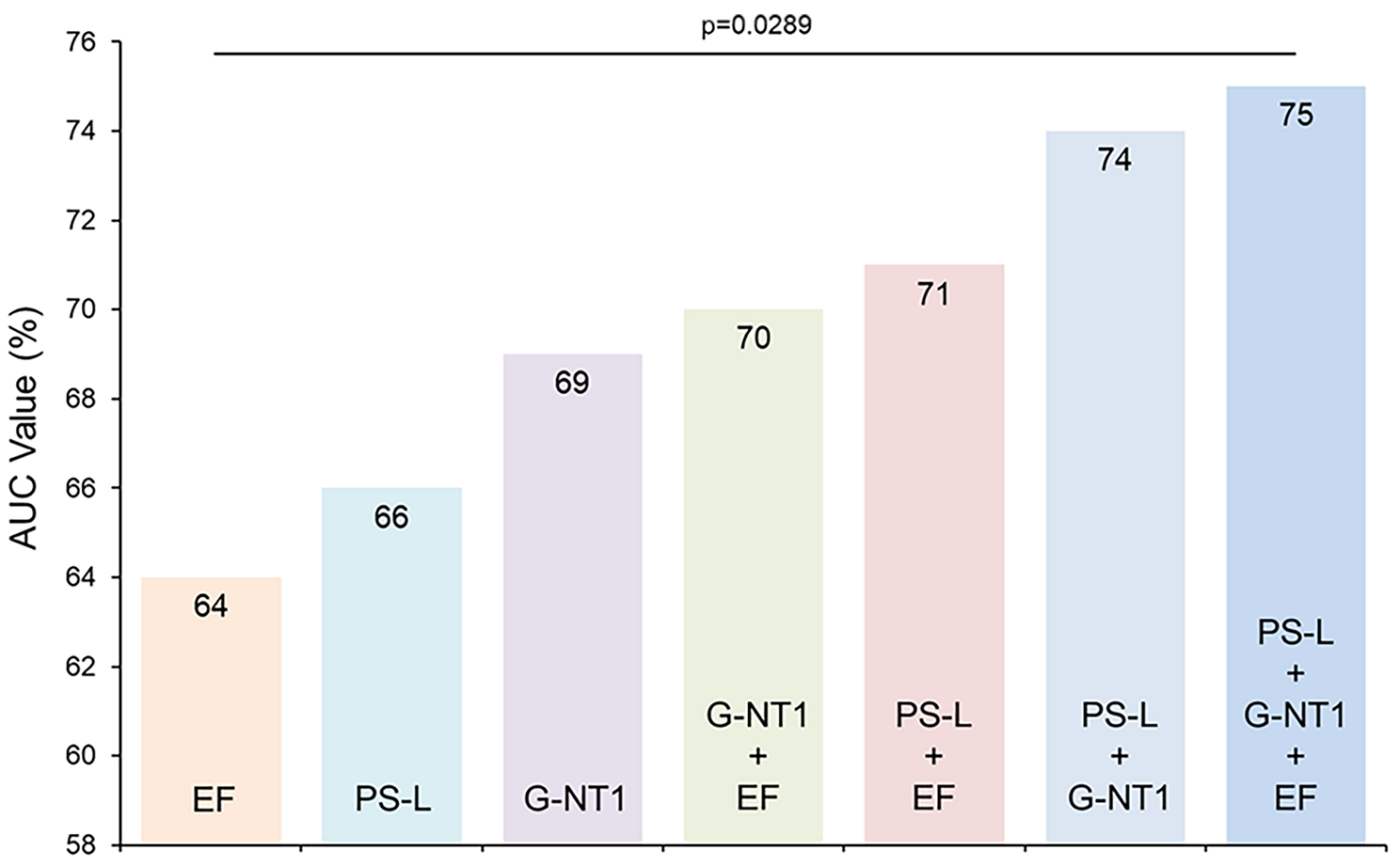
AUC comparisons of different models

EF, global native T1 value and PS-L were used as LGE prediction parameters, respectively. Furthermore, regression analysis was performed in a forward direction for the combination of these three LGE related indicators, producing the following other four prediction model equations: model 4 = − 8.92 − 0.061*EF + 0.01*global native T1; model 5 = 7.478 − 0.066*EF − 0.231* PS-L; model 6 = − 10.822 + 0.011* global native T1 − 0.241*PS-L; and model 7 = − 5.639 − 0.051*EF + 0.009* global native T1 − 0.223*PS-L. The three parameters EF, global native T1 value, PS-L and the four combined equations constituted a total of seven models. The AUC for each of the seven prediction models were 0.64, 0.69, 0.66, 0.70, 0.71, 0.74 and 0.75 (Fig. 2). The threshold, sensitivity, specificity and other indexes of the different models are shown in Table 4. Among them, the sensitivity of the PS-L model was the highest (77.78%), the specificity of model 4 was the best (90.20%) and the sensitivity and specificity of model 5, 6 and 7 were all above 70%. For the seven models, the prediction thresholds of LGE were 58.95%, − 16.94%, 1303.12 ms, 0.68, − 0.06, − 0.50 and − 0.63, respectively. The AUC for model 7 was the highest (0.75). Although AUC from model 1 to 7 increased gradually, except for EF and model 7 (0.64 vs. 0.75, p = 0.029), there was no statistically significant difference in the AUC among the other models (Fig. 3). However, in the < 10 years group, the PS-L cannot well distinguish whether LGE occurs (p > 0.05); And in the ≥ 10 years group, global native T1 and the combined index of native T1 and PS-L can distinguish LGE well, but the difference between the two models was not statistically significant (see Additional file 1: Supplementary Fig. 1).

**Table 4:** Threshold performance for models incorporating EF, PS-Longitudinal and Global Native T1

Model	AUC	Threshold	Sensitivity%	Specificity%	+LR	-LR
**EF**	0.64	58.95	55.56	70.59	1.89	0.63
**PS-Longitudinal**	0.66	−16.94	77.78	49.02	1.53	0.45
**Global Native T1**	0.69	1303.12	57.78	86.27	4.21	0.49
−**8.92** − 0.061*EF + 0.01*Global Native T1	0.70	0.68	51.11	90.20	5.21	0.54
**7.478** − 0.066*EF − 0.231* PS-Longitudinal	0.71	−0.06	73.33	70.59	2.49	0.38
−10.822 + 0.011*Global Native T1 − 0.241*PS-Longitudinal	0.74	−0.50	73.33	70.59	2.49	0.38
−**5.639** − 0.051*EF + 0.009*Global Native T1 − 0.223*PS-Longitudinal	0.75	−0.63	73.33	74.51	2.88	0.36

+LR: Positive likelihood ratio; -LR: Negative likelihood ratio

### Validation cohort

In the validation cohort, compared with the LGE − group, the global native T1 value in the LGE + group increased (1301.83 ± 41.97 vs. 1269.73 ± 41.77, p = 0.021), while the absolute value of PS-L decreased (− 15.16 ± 2.20 vs. −17.08 ± 2.09, p = 0.007). And the basic demographic characteristics as well as the three pre-contrast parameters in the multivariate logistic regression of the validation cohort are shown in Additional file 1: Supplementary Table 2.

Table 5 provides a summary of the prediction performance of all models. Among them, the single PS-L index prediction model had the highest sensitivity of 83.33%, but the lowest specificity of 54.55%, whereas the single global native T1 index prediction model had the best specificity of 95.45%, but the lowest sensitivity of 38.89%. Besides, the sensitivity and specificity of the other multi-parameter models were moderate. The results of using the thresholds for each model derived from the derivation cohort to predict the LGE status of patients in the validation cohort are shown in Additional file 1: Supplementary Table 3. Although models 1, 3, 5, 6 and 7 were consistent with the diagnosis, the agreement was mild to moderate (Kappa values were 0.22–0.40). The consistency of model prediction and diagnosis outcomes are shown in Additional file 1: Supplementary Table 4.

**Table 5:** Prediction performance of all models in validation cohort

	Sensitivity%	Specificity%	+PV	-PV	Youden index
**EF**	44.45	77.27	61.54	62.96	0.22
**Global Native T1**	38.89	95.45	87.50	65.63	0.34
**PS-Longitudinal**	83.33	54.55	60.00	80.00	0.38
**EF & Global Native T1**	33.33	90.91	75.00	62.50	0.24
**EF & PS-Longitudinal**	55.56	68.18	58.82	65.22	0.24
**Global Native T1 & PS-Longitudinal**	72.22	68.18	65.00	65.00	0.41
**EF & Global Native T1 & PS-Longitudinal**	72.22	68.18	65.00	65.00	0.41

+PV: Positive predictive value; -PV: Negative predictive value

## Discussion

Our study found that pre-contrast sequence parameters, including LVEF, native T1 and global longitudinal strain, may be independent predictors for LGE presence in DMD patients. Based on the three parameters, we derived different linear regression models. In the derivation cohort, the model combining the EF, native T1 and longitudinal strain exhibited the best diagnostic value, pre-contrast sequences offer a moderate predictive value for the presence of LGE. Compared with single index models, composite multi-parameter regression models had increased diagnostic efficiency, but not statistically significantly. In addition, we introduced the validation cohort for the first time in such studies of pre-contrast sequence performance to predict LGE in DMD patients. In the validation cohort, we further confirmed that these three parameters and their composite regression models can moderately predict LGE for individual CMRs, and good sensitivity can be obtained by using global longitudinal strain only. Furthermore, the performance of these models derived from the pre-contrast sequence parameter was mediocre, suggesting that non-contrast sequence imaging in DMD patients could not completely replace LGE imaging. Nonetheless, these models might be applied when the GBCAs CMR was contradicted and could not be finished for the reasons stated in the introduction.

Different from previous studies, we included models that incorporated different parameters through multivariable regression screening and compared the predictive efficiency to assess whether fewer, simpler CMR parameters might be employed as a substitute for LGE. Compared with the segmental CMR parameters patterns demonstrated by Raucci Jr. and colleagues [18], the results of the analyses of global parameters showed similar predictive performance, and the global data were easier to obtain in application. At the same time, concerning about the safety of using GBCAs in younger children, the age range of our subjects was also lower than that of the patients enrolled in the aforementioned research, and it was found that the pre-contrast models also had a moderate predictive ability for the occurrence of LGE in younger DMD children. The application of pre-contrast models might have more clinical significance given the characteristics that younger children are more difficult to cooperate with. More crucially, we applied the results from the derivation cohort to the validation cohort, which further verified the accuracy and applicability of the model and boosted the reliability of our findings.

In our study, the performance of multi-parameter models, such as LVEF and global native T1, LVEF and longitudinal strain, in predicting LGE had not been significantly improved. EF and global native T1 primarily reflect global myocardial abnormalities, while previous studies have demonstrated that myocardial fibrotic change in individuals with DMD mostly begins and focuses on the lateral free wall, which is typically patchy [24–26]. The discrepancy between global LV function and myocardial tissue indexes and focal LGE distribution might be the reason for the reduced consistency between the predicted results of pre-contrast models and the actual presence of LGE. Until late in the illness, cardiac function in DMD patients is generally normal. Despite the beginning of myocardial fibrosis, LVEF still remain normal in many patients [7]. The appearance of fibrosis often precedes the change of LVEF [27]; therefore, LVEF and models including EF have good specificity but poor sensitivity in predicting LGE. The pathophysiological alterations in muscle in DMD patients are mainly fatty infiltration and fibrosis, which have been well documented in the skeletal muscle [28]. Increased fatty infiltration in cardiac tissue might result in lower native T1 value [29]; hence, several factors may influence the relationship between native T1 value and LGE in DMD patient. The proportion of fat content may lead to the poor sensitivity of the prediction models that include native T1 value. In addition, our study found that global T2 values did not change significantly in LGE subgroups of DMD patients and might not be predictive of LGE. Also, based on the results of the different age group, in the group under 10 years old, longitudinal strain had shown a tendency to predict LGE, and in the group over 10 years old, due to the aggravation of myocardial injury, the corresponding pre-contrast parameters’ changes such as native T1 and longitudinal strain were more obvious, so the prediction effect of the pre-contrast sequences models was better than that of the group under 10 years old in the derivation cohort. We believed that in the early stage of this disease, most myocardial abnormalities are first manifested in parameters such as myocardial strain that reflect early systolic and diastolic function. With the increase of age, the myocardial damage of DMD patients is aggravated, normal myocardial tissue is replaced by connective tissue and adipose tissue, and the degree of myocardial fibrosis is also aggravated. In addition to focal fibrosis, diffuse fibrosis occurs in the myocardium, and has a certain correlation with the occurrence of LGE.

Interestingly, compared with the multi-parameter models, single-index prediction models, such as longitudinal strain, had better sensitivity in the prediction of LGE. According to several studies, myocardial longitudinal strain decreases sooner in the early stage of DMD, and has a good correlation with myocardial injury, including myocardial fibrosis and heart failure [30, 31]. In addition, although some models, such as native T1, were not sensitive enough to predict LGE, they had high specificity. Native T1 generally does not increase significantly in the absence of focal or diffuse fibrosis in the myocardium [32], giving native T1 a superior performance in excluding DMD patients with LGE. Regarding the suboptimal results of most multi-parameter models, we thought that in patients with absolute contraindications to GBCAs, with no intravenous access or those who refuse the use of GBCAs, the single-index model-longitudinal strain can help evaluate whether these patients are likely to develop LGE and then introduce the drug intervention in time. And DMD patients who may not have LGE might also be excluded with the use of native T1 value. Compared to multi-parameter models, single-index models make LGE prediction simpler and easier to implement in primary medical centers with limited access to mapping sequences and professional post-process tools.

Despite predictive results in the validation cohort being unsatisfactory, these models are still important in DMD patients. DMD patients will find it challenging to maintain supine postures for long periods of examination time because of severe dyskinesia, scoliosis and respiratory dysfunction caused by progressive muscle damage [33, 34]. In addition, the patient’s breath-holding capacity is limited by progressive respiratory weakness [35]. These reasons may lead to the decline of the patient’s image quality. Reducing scanning time is a significant consideration to enhance patient compliance, image quality and examination safety. As eliminating contrast administration can generally shorten the scanning time by a quarter, the pre-contrast prediction models could be considered for patients with poor compliance because of physical limitations or respiratory disorder.

### Limitations

There are some limitations of our study. First, the derivation and validation cohorts introduced in the study were not real machine learning, but rather basic manual verification. Given the rarity of DMD incidence and the long time it took to recruit enough DMD participants in a single center, the small number of instances may contribute to the low prediction accuracy and the small number of cases in our validation cohort cannot support machine learning with a large sample size. However, our results had shown some predictive trends and value in the relatively small sample, so in subsequent studies, we will expand the number of examples and consider applying artificial intelligence machine learning for prediction model research. Second, this was a single-centre design study, with some bias in case selection and a lack of prospective longitudinal evaluation of the occurrence of LGE. Third, given the focal patchy manifestations of fibrosis in DMD myocardial damage, segmental pre-contrast parameters may be more predictive in areas where LGE is commonly observed [36]. Instead of segmental indexes, we employed global parameters in our study. Further explorations will be performed on the LGE predictive performance of pre-contrast sequences based on segmental data of strain, native T1 and other parameters.

## Conclusions

Through the derivation and validation cohorts, we considered that pre-contrast sequences models had moderate predictive value for the presence of LGE in young DMD patients. Although the predictive efficacy of models were restricted, longitudinal strain might be used as a surrogate for LGE in DMD patients with absolute contraindications to GBCAs, no intravenous access and poor compatibility requiring reduced examination time. However, given the critical information that LGE imaging can provide, non-contrast imaging protocol still cannot completely replace LGE imaging at present, and DMD patients without exceptional circumstances should continue to have standard gadolinium-enhanced CMR scanning performed regularly.

### Electronic supplementary material

Below is the link to the electronic supplementary material.

Supplementary Material 1: Supplementary Table 1: Baseline characteristics of the derivation (n = 96) and validation cohorts (n = 40);

Supplementary Material 2: Supplementary Table 2: Baseline characteristics and partial CMR parameters in validation cohort;

Supplementary Material 3: Supplementary Table 3: Comparison between model diagnosis results and imaging diagnosis results; Supplementary Table 4: The consistency of model prediction and diagnosis results

## Data Availability

The datasets used and analysed during the current study are not publicly available due to the subsequent relevant research has not been completed but are available from the corresponding author on reasonable request.

## Abbreviations

CMR: Cardiovascular magnetic resonance
LGE: Late gadolinium enhancement
DMD: Duchenne muscular dystrophy
LV: Left ventricle
GBCAs: Gadolinium-based contrast agents
PS-L: Peak strain-longitudinal
PS-C: Peak strain-circumferential
PS-R: Peak strain-radial
EF: Ejection fraction
EDV: End-diastolic volume
ESV: End-systolic volume
OR: Odd ratio
CI: Confidence interval
ROC: Receiver operating characteristic
AUC: Area under curves
BSA: Body surface area
LVGFI: LV global function index
