# Supplementary material for: Derivation and validation of diagnostic models for myocardial fibrosis in duchenne muscular dystrophy: assessed by multi-parameter cardiovascular magnetic resonance
Source: Orphanet J Rare Dis. 2023 Dec 11;18:388. doi: 10.1186/s13023-023-02931-y (PMC10714650; doi:10.1186/s13023-023-02931-y)
Supplement: Supplementary file 1 — Supplementary Material 1: Supplementary Table 1: Baseline characteristics of the derivation (n = 96) and validation cohorts (n = 40); [file 13023_2023_2931_MOESM1_ESM.docx]

**Supplementary Table 1** Baseline characteristics of the derivation (n = 96) and validation cohorts (n = 40)

|  | **Derivation**  **N =96** | **Validation**  **N =40** | **P**  **value** |
| --- | --- | --- | --- |
| ***Baseline characteristics*** |  |  |  |
| **Age, years** | 8.72±2.27 | 9.14±1.33 | 0.278 |
| **Height, cm** | 126.84±12.33 | 123.60±7.63 | 0.125 |
| **Weight, kg** | 28.82±9.35 | 27.71±6.82 | 0.498 |
| **BMI, kg/m^2^** | 17.55±3.57 | 17.97±3.17 | 0.517 |
| **BSA, m^2^** | 0.99±0.19 | 0.96±0.12 | 0.289 |
| **Heart rate, bpm** | 96.55±14.51 | 96.25±12.58 | 0.909 |
| ***Medications*** |  |  |  |
| **Corticosteroids** | 68 (70.83) | 31(75.00) | 0.426 |
| **ACEI** | 17 (17.71) | 7(17.50) | 0.977 |
| **β-blocker** | 17 (17.71) | 7(17.50) | 0.977 |
| **Diuretic** | 6 (6.25) | 2(5.00) | 0.778 |

Values are presented as mean ± standard deviation or n (%).

**Supplementary Table 2** Univariable and multivariable logistic regression for the association between LGE and baseline and CMR variables in patients <10 years

|  | **Univariable OR**  **(95% CI)** | **P**  **value** | **Multivariable OR**  **(95% CI)** | **P**  **value** |
| --- | --- | --- | --- | --- |
| **Age** | 1.44（0.93-2.23） | 0.10§ | 1.58（0.91-2.76） | 0.10 |
| **BMI** | 1.19（0.99-1.42） | 0.07§ | 1.23（0.96-1.58） | 0.10 |
| **HR** | 1.00（0.97-1.03） | 0.91 |  |  |
| **EF** | 0.93（0.86-1.00） | 0.05* | 0.92（0.75-1.14） | 0.46 |
| **LV remodeling index** | 0.25（0.00-55.48） | 0.61 |  |  |
| **LVGFI** | 0.94（0.88-1.01） | 0.09§ | 0.99（0.82-1.19） | 0.92 |
| **Global Native T1** | 1.019（1.00-1.02） | 0.11 |  |  |
| **T2** | 1.12（0.92-1.35） | 0.25 |  |  |
| **PS-Radial** | 0.89（0.79-1.01） | 0.06§ | 0.98（0.81-1.19） | 0.87 |
| **PS-Circumferential** | 0.80（0.66-0.98） | 0.03* | 0.83（0.60-1.16） | 0.28 |
| **PS-Longitudinal** | 0.81（0.65-1.00） | 0.05* | 0.75（0.57-0.98） | 0.04* |

§p＜0.1

*p＜0.05

** p＜0.01

**Supplementary Table 3** Univariable and multivariable logistic regression for the association between LGE and baseline and CMR variables in patients ≥10 years

|  | **Univariable OR**  **(95% CI)** | **P**  **value** | **Multivariable OR**  **(95% CI)** | **P**  **value** |
| --- | --- | --- | --- | --- |
| **Age** | 0.96（0.56-1.65） | 0.89 |  |  |
| **BMI** | 0.86（0.69-1.09） | 0.22 |  |  |
| **HR** | 1.03（0.97-1.09） | 0.38 |  |  |
| **EF** | 0.93（0.84-1.02） | 0.12 |  |  |
| **LV remodeling index** | 0.04（0.00-12.16） | 0.26 |  |  |
| **LVGFI** | 0.97（0.90-1.05） | 0.45 |  |  |
| **Global Native T1** | 1.04（1.01-1.07） | 0.01** | 1.05（1.01-1.09） | 0.01** |
| **T2** | 0.86（0.64-1.15） | 0.30 |  |  |
| **PS-Radial** | 0.94（0.79-1.13） | 0.52 |  |  |
| **PS-Circumferential** | 0.93（0.67-1.27） | 0.64 |  |  |
| **PS-Longitudinal** | 0.73（0.53-1.02） | 0.07§ | 0.56（0.34-0.92） | 0.02* |

§p＜0.1

*p＜0.05

** p＜0.01

**Supplementary Table 4** Baseline characteristics and partial CMR parameters in validation cohort

|  | **DMD LGE +**  **N =18** | **DMD LGE−**  **N =22** | **P**  **value** |
| --- | --- | --- | --- |
| **Age, years** | 9.58±1.24 | 8.77±1.32 | 0.054 |
| **Height, cm** | 126.22±8.98 | 121.45±5.66 | 0.047* |
| **Weight, kg** | 29.16±7.54 | 26.52±6.09 | 0.229 |
| **EF, %** | 58.57±6.98 | 61.79±5.01 | 0.097 |
| **Global Native T1, ms** | 1301.83±41.97 | 1269.73±41.77 | 0.021* |
| **PS-Longitudinal (%)** | −15.16±2.20 | −17.08±2.09 | 0.007** |

*p＜0.05

** p＜0.01

Values are presented as mean ± standard deviation.

**Supplementary Table 5** Comparison between model diagnosis results and imaging diagnosis results

|  |  | **Diagnostic result** | |  |
| --- | --- | --- | --- | --- |
| **Model** |  | **LGE**− | **LGE+** | **Total** |
| **EF** | **LGE**− | 17 | 10 | 27 |
|  | **LGE+** | 5 | 8 | 13 |
|  |  |  |  |  |
| **Global Native T1** | **LGE**− | 21 | 11 | 32 |
|  | **LGE+** | 1 | 7 | 8 |
|  |  |  |  |  |
| **PS-Longitudinal** | **LGE**− | 12 | 3 | 15 |
|  | **LGE+** | 10 | 15 | 25 |
|  |  |  |  |  |
| **EF & Global Native T1** | **LGE**− | 20 | 12 | 32 |
|  | **LGE+** | 2 | 6 | 8 |
|  |  |  |  |  |
| **EF & PS-Longitudinal** | **LGE**− | 15 | 8 | 23 |
|  | **LGE+** | 7 | 10 | 17 |
|  |  |  |  |  |
| **Global Native T1 & PS-Longitudinal** | **LGE**− | 15 | 5 | 20 |
|  | **LGE+** | 7 | 13 | 20 |
|  |  |  |  |  |
| **EF & Global Native T1 & PS-Longitudinal** | **LGE**− | 15 | 5 | 20 |
|  | **LGE+** | 7 | 13 | 20 |
|  |  |  |  |  |
| **Total** |  | 22 | 18 |  |

**Supplementary Table 6** The consistency of model prediction and diagnosis results

|  | **Kappa** | **P value** |
| --- | --- | --- |
| **EF** | 0.22 | 0.30 |
| **Global Native T1** | 0.36 | 0.01 |
| **PS-Longitudinal** | 0.37 | 0.09 |
| **EF & Global Native T1** | 0.26 | 0.01 |
| **EF & PS-Longitudinal** | 0.24 | 1.00 |
| **Global Native T1 & PS-Longitudinal** | 0.40 | 0.77 |
| **EF & Global Native T1 & PS-Longitudinal** | 0.40 | 0.77 |


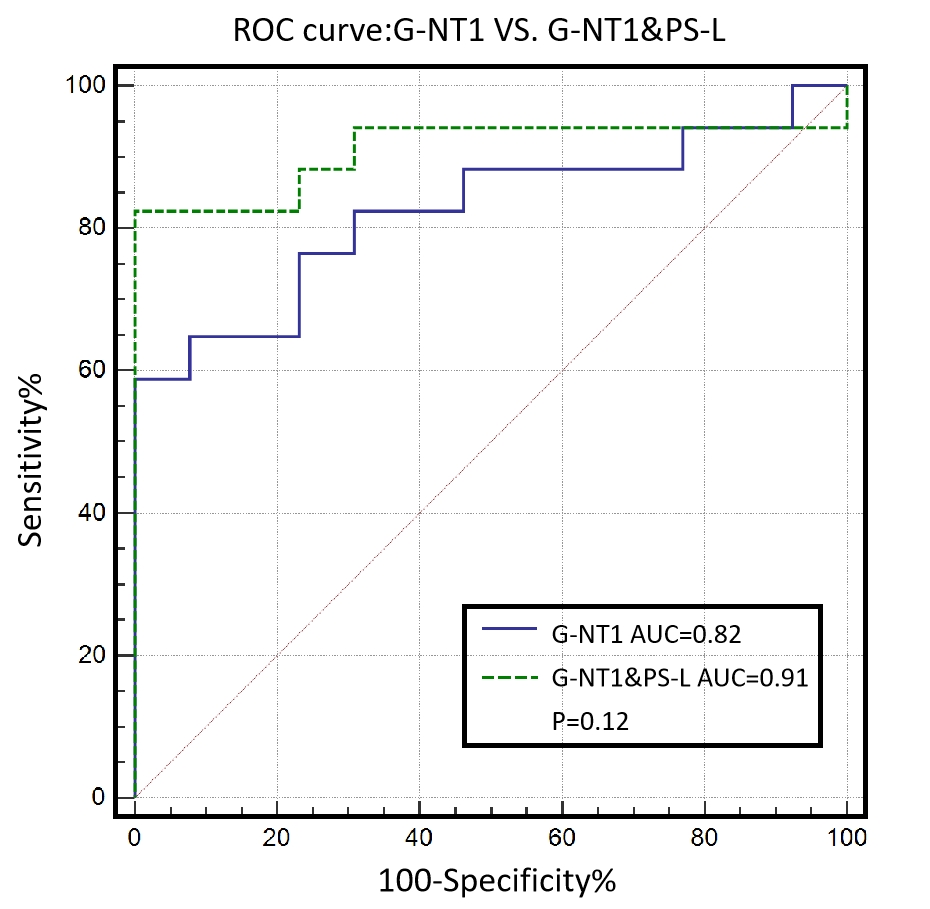


**Supplementary Figure 1** AUC comparisons of Global native T1 & Global native T1+PS-Longitudinal
