# Supplementary material for: Derivation and validation of diagnostic models for myocardial fibrosis in duchenne muscular dystrophy: assessed by multi-parameter cardiovascular magnetic resonance
Source: Orphanet J Rare Dis. 2023 Dec 11;18:388. doi: 10.1186/s13023-023-02931-y (PMC10714650; doi:10.1186/s13023-023-02931-y)
Supplement: Supplementary file 2 — Supplementary Material 2: Supplementary Table 2: Baseline characteristics and partial CMR parameters in validation cohort; [file 13023_2023_2931_MOESM2_ESM.docx]

**Derivation and validation of diagnostic models for myocardial fibrosis in Duchenne muscular dystrophy： assessed by multi-parameter cardiovascular magnetic resonance**

Zi-qi Zhou ^1^, Hua-yan Xu ^1^, Hang Fu ^1^, Ke Xu ^1^, Rong Xu ^1^, Xiao-tang Cai ^2*^ and Ying-kun Guo ^1*^

^1^ Department of Radiology, Key Laboratory of Birth Defects and Related Diseases of Women and Children of Ministry of Education, West China Second University Hospital, Sichuan University, 20# Section 3 South Renmin Road, Chengdu, 610041, China.

^2^ Department of Pediatrics Neurology, West China Second University Hospital, Sichuan University, 20# Section 3 South Renmin Road, Chengdu 610041, China

^*^ Xiao-tang Cai and Ying-kun Guo contributed equally to this work and should be considered as co-corresponding authors.

**Guarantor and correspondent:**

**Xiao-tang Cai**

Department of Pediatrics Neurology, West China Second University Hospital, Sichuan University, 20# Section 3 South Renmin Road, Chengdu 610041, China

E-mail: [cxt_1999@126.com](mailto:cxt_1999@126.com)

**Ying-kun Guo**

Department of Radiology, Key Laboratory of Birth Defects and Related Diseases of Women and Children of Ministry of Education, West China Second University Hospital, Sichuan University; 20# Section 3 South Renmin Road, Chengdu, 610041, China

Tel: +86-28-85503275(O) E-mail: gykpanda@163.com

**Email addresses of all co-authors:**

Zi-qi Zhou: 945849257@qq.com

Hua-yan Xu: xuhuayan89@sina.com

Hang Fu: fuhang66666@163.com

Ke Xu: [kexu0302@163.com](mailto:kexu0302@163.com)

Rong Xu: [Xrongdoctor@163.com](mailto:Xrongdoctor@163.com)

Xiao-tang Cai: [cxt_1999@126.com](mailto:cxt_1999@126.com)

Ying-kun Guo: [gykpanda@163.com](mailto:gykpanda@163.com)

**Study design:**prospective,single-centre design and diagnostic study
