# Supplementary material for: Derivation and validation of diagnostic models for myocardial fibrosis in duchenne muscular dystrophy: assessed by multi-parameter cardiovascular magnetic resonance
Source: Orphanet J Rare Dis. 2023 Dec 11;18:388. doi: 10.1186/s13023-023-02931-y (PMC10714650; doi:10.1186/s13023-023-02931-y)
Supplement: Supplementary file 3 — Supplementary Material 3: Supplementary Table 3: Comparison between model diagnosis results and imaging diagnosis results; Supplementary Table 4: The consistency of model prediction and diagnosis results [file 13023_2023_2931_MOESM3_ESM.docx]

Dear Prof. Elizabeth A. BRAUNLIN,

Sincerely thanks for the questions, recommendations and positive comments from the reviewers. Here we submit our latest revised manuscript entitled "Derivation and validation of diagnostic models for myocardial fibrosis in Duchenne muscular dystrophy： assessed by multi-parameter cardiovascular magnetic resonance" to Orphanet Journal of Rare Diseases at the online submission system.

According to the recommendations of reviewers, we have carefully amended the manuscript by using the ‘track changes’ option and made a point-to-point respond to these questions. We made this revision on the final version of the manuscript after the last revision. Revision in the manuscript was marked in red. The other changes were underlined. We replaced the manuscript submitted before and re-uploaded the latest revised manuscript.

All of the authors have read and approved the final revised version. We hope the manuscript can go with your expectation for publication in Orphanet Journal of Rare Diseases.

**Responses to reviewers:**

**Reviewer #1:**

I read your article with interest. I think always determining other ways to identify presence of gadolinium enhancement are always of use in several patient physiologies.
**1.** Can you please further describe strain from MRI measurement? Most cardiologists understand it from echocardiography, but please outline it further in the methods section.

**Response:** Thanks for your suggestion, we have added a simple explanation in the methods section. The post-processing method for obtaining myocardial strain parameters using echocardiography is mainly based on the speckle tracking technique, which evaluates myocardial strain by tracking the movement trajectory of the myocardium [1]. In this study, myocardium strain parameters of myocardium were obtained based on cine images using CMR SSFP sequences. By delineating the endocardium and epicardium in diastole in the tissue feature tracking module of the post-processing software, relative motion and displacement of the myocardium voxels in the cardiac cycle were automatically tracked, so as to obtain the myocardial strain parameters, which is basically similar to the principle of myocardial strain measurement by echocardiography. And the longitudinal, radial and circumferential strain parameters can be obtained according to the different directions of myocardial movement.

**2.** Regarding Kappa, please describe how good the test cohort Kappa values truly were. How good is 0.4?

**Response：**Generally, it is believed in statistics that a Kappa value less than 0.2 indicates poor consistency between results, 0.2-0.4 indicates mild consistency, 0.4-0.6 indicates moderate consistency, and 0.6-0.8 indicates good consistency. In the validation cohort, the consistency between the predictive ability of our models and the actual occurrence of LGE was mild to moderate, with kappa values ranging from 0.22 to 0.4, and the predictive results were not satisfactory. In the discussion section, we have explained this result and analyzed the possible reasons for the lack of good consistency.

**3.** In the discussion section please describe further the significant of difference between the T1 and T2 signals you saw between those with gadolinium versus those without gadolinium enhancement. We're you expecting this difference?

**Response:** Thanks for your suggestion, we have briefly added description in the discussion section. Our results showed that global native T1 value significantly increased in LGE+ patient, whereas T2 values did not. This difference in the results of different signals may be due to the characteristics of cardiac pathophysiological changes in patients. In DMD patients of this age, it has been indicated that the occurrence of focal or diffuse fibrosis can lead to an increase in T1 value [2]. And we have another in writing research on T2 signal changes in DMD patients, which found that the global T2 value changes in patients were not significant. This may be due to the overall myocardial edema and inflammation level of patients is still in an early stage, and we will continue to pay attention to the changes in the T2 signal in subsequent studies.

**4.** For further direction, should other modality parameters be included in the model if a subsequent model were to be made?

**Response:** Thank you for your inspiration. In the limitation section, we considered further optimizing these models by adding myocardial segment parameters in the future study to improve models' prediction ability. Meanwhile, with the development of magnetic resonance technology and machine learning, more refined parameters such as myocardial oxygen content obtained by magnetic resonance blood oxygenation level dependent technology and texture features obtained by radiomics are expected to be added to the establishment of new prediction models. We believe that based on the existing universal technical means, the prediction models in our paper also had certain clinical application value.

**References**

1. Barbosa JA, Mota CC, Simões E Silva AC, Nunes Mdo C, Barbosa MM. Assessing pre-clinical ventricular dysfunction in obese children and adolescents: the value of speckle tracking imaging. Eur Heart J Cardiovasc Imaging. 2013;14(9):882-889

2. Xu K, Xu HY, Xu R, Xie LJ, Yang ZG, Yu L, et al. Global, segmental and layer specific analysis of myocardial involvement in Duchenne muscular dystrophy by cardiovascular magnetic resonance native T1 mapping. J Cardiovasc Magn Reson. 2021;23(1):110.

**Reviewer #2:**

The aim of the stsudy is indeed very interesting however there are several methodological issues which limit the interpretability of this search for an alternative diagnostic to measure cardiomyopathy.

The first is the patient population which was included. It was noted patients included in this study were on average younger than when the myopathy starts to occur which is in older patients. Here we have an average of 8yrs in a paper by Hor et al. Journal of Cardiovascular Magnetic Resonance 2013, 15:107, there are several considerations which are important in determining the usefulness in using LGE. One is age as cardiomyopathy associated with fibrosis occurs in older patients. this does not seem to be accounted for in this study. Secondly the predictive nature is associated with a LVEF <55% also not discussed in the authors paper nor in the methodology. Finally in this paper it is noted that " DMD subjects showed LGE positivity with prevalence increasing from 17% of patients <10 years to 34% of those aged 10-15 years and 59% of those >15 years-old. Patients with LVEF ≥55% were LGE positive in 30% of cases; this increased to 84% for LVEF <55%. LGE was more prevalent in the free wall (531/1243, 42.7%) vs. septal segments (30/565, 5.3%). Patients with septal involvement were significantly older and had lower LVEF than those with isolated free wall LGE." The authors seem to assume that the cardiomyopathy is stable and group everything together. The statistical anaylsis of this study will not I my opinion help much because there is no stratification taken into consideration to account for the variability in age. We have no idea of the capacity to differentiate by age group of the alternative diagnostic approach. The authors should do additional analysis regarding age and LVEF by age group which might help the reader understand if the alternative diagnostic approach is really modest or not. In the opinion of this reviewer more consideration needs to be given to the underlying cardiomyopathy and age. This needs to be factored in to the statistical analysis.

**Response:** Thank you for your suggestion, it is very enlightening for us. With the advancement of DMD diagnosis, deepening understanding of DMD related myocardial injury and the development of imaging technology, more and more young DMD patients are starting to use CMR for myocardial assessment. However, due to the poor compliance of younger patients and the high risk of contrast agent allergy for them, we wanted to explore whether the pre-contrast sequences can effectively predict the occurrence of LGE under the trend of gradually younger myocardial damage found in DMD patients. Therefore, the overall age level of the subjects included in this study was younger than in previous studies. Meanwhile, due to the younger age of the included patients, the LVEF of most patients was still at a normal level, that is, LVEF ≥55%. Among the 96 patients in the derivation cohort of this study, only 10 had an LVEF < 55%. In discussion section, we also explained this phenomenon.

The cardiomyopathy of DMD patients progresses with age. Based on your suggestion, we divided the 96 patients in derivation cohort into groups under 10 years old (66 participants) and ≥10 years old (30 participants). In the group under 10 years old, 23 had LGE, only longitudinal strain was an independent risk factor for the occurrence of LGE , but its ability to predict LGE was relatively poor. In addition to longitudinal strain, global native T1 value was also an independent risk factor for LGE and can predict the occurrence of LGE in the group over 10 years. At the same time, the new model composed of above two parameters, model 3 = -54.611+0.05*native T1 value-0.586*PS-L also had predictive value for the occurrence of LGE, but there was no statistical significance in its predictive ability compared with native T1 value. We believed that in the early stage of this disease, most myocardial abnormalities are first manifested in parameters such as myocardial strain that reflect early systolic and diastolic function, and the changes in myocardial strain is not synchronized with the occurrence of myocardial fibrosis sometimes. With the increase of age, the myocardial damage of DMD patients is aggravated, normal myocardial tissue is replaced by connective tissue and adipose tissue, and the degree of myocardial fibrosis is also aggravated. In addition to focal fibrosis, diffuse fibrosis occurs in the myocardium, and has a certain correlation with the occurrence of LGE. In the group under 10 years old, PS-L had shown a tendency to predict LGE, although it did not reach statistical significance. In the group over 10 years old, due to the aggravation of myocardial injury, the corresponding pre-contrast parameters’ changes were more obvious, so the prediction effect of the pre-contrast sequences models was better than that of the group under 10 years old in the derivation cohort. However, because the small number of patients included in the validation cohort, with only 12 patients ≥10 years old, the effect of these models applied to the validation cohort was unsatisfactory, and the small sample size also led to inaccurate and unreliable statistical results. We explained this point further in limitation section. The results by age group were generally consistent with the existing results in our study, suggesting that the partial pre-contrast parametric models might be useful in children older than 10 years, but the non-contrast imaging protocol still cannot completely replace LGE imaging at present, and DMD patients without special conditions should continue to undergo regular standard gadolinium-enhanced CMR scanning. Part of the new statistical analysis and results were described in the article and additional file.

In addition, according to your suggestion, we tried to group the derivation and validation cohort according to the threshold of LVEF=55%. However, since the cardiac function of most patients has not been significantly reduced, and the number of patients with LVEF < 55% was small, the statistical results were not satisfactory. We would track the changes in patients' cardiac function in the subsequent studies. (All new statistical analysis results were presented in the response to reviewers)

**Table 1** Univariable and multivariable logistic regression for the association between LGE and baseline and CMR variables in patients <10 years

|  | **Univariable OR**  **(95% CI)** | **P**  **value** | **Multivariable OR**  **(95% CI)** | **P**  **value** |
| --- | --- | --- | --- | --- |
| **Age** | 1.44（0.93-2.23） | 0.10§ | 1.58（0.91-2.76） | 0.10 |
| **BMI** | 1.19（0.99-1.42） | 0.07§ | 1.23（0.96-1.58） | 0.10 |
| **HR** | 1.00（0.97-1.03） | 0.91 |  |  |
| **EF** | 0.93（0.86-1.00） | 0.05* | 0.92（0.75-1.14） | 0.46 |
| **LV remodeling index** | 0.25（0.00-55.48） | 0.61 |  |  |
| **LVGFI** | 0.94（0.88-1.01） | 0.09§ | 0.99（0.82-1.19） | 0.92 |
| **Global Native T1** | 1.019（1.00-1.02） | 0.11 |  |  |
| **T2** | 1.12（0.92-1.35） | 0.25 |  |  |
| **PS-Radial** | 0.89（0.79-1.01） | 0.06§ | 0.98（0.81-1.19） | 0.87 |
| **PS-Circumferential** | 0.80（0.66-0.98） | 0.03* | 0.83（0.60-1.16） | 0.28 |
| **PS-Longitudinal** | 0.81（0.65-1.00） | 0.05* | 0.75（0.57-0.98） | 0.04* |

§p＜0.1

*p＜0.05

** p＜0.01

**Table 2** Univariable and multivariable logistic regression for the association between LGE and baseline and CMR variables in patients ≥10 years

|  | **Univariable OR**  **(95% CI)** | **P**  **value** | **Multivariable OR**  **(95% CI)** | **P**  **value** |
| --- | --- | --- | --- | --- |
| **Age** | 0.96（0.56-1.65） | 0.89 |  |  |
| **BMI** | 0.86（0.69-1.09） | 0.22 |  |  |
| **HR** | 1.03（0.97-1.09） | 0.38 |  |  |
| **EF** | 0.93（0.84-1.02） | 0.12 |  |  |
| **LV remodeling index** | 0.04（0.00-12.16） | 0.26 |  |  |
| **LVGFI** | 0.97（0.90-1.05） | 0.45 |  |  |
| **Global Native T1** | 1.04（1.01-1.07） | 0.01** | 1.05（1.01-1.09） | 0.01** |
| **T2** | 0.86（0.64-1.15） | 0.30 |  |  |
| **PS-Radial** | 0.94（0.79-1.13） | 0.52 |  |  |
| **PS-Circumferential** | 0.93（0.67-1.27） | 0.64 |  |  |
| **PS-Longitudinal** | 0.73（0.53-1.02） | 0.07§ | 0.56（0.34-0.92） | 0.02* |

§p＜0.1

*p＜0.05

** p＜0.01

**Figure 1 AUC comparisons of Global native T1 & Global native T1+PS-Longitudinal**

**Table 3** Threshold performance for models incorporating Global Native T1 and PS-Longitudinal in patients ≥10 years

| **Model** | **AUC** | **Threshold** | **Sensitivity%** | **Specificity%** | **+LR** | **-LR** |
| --- | --- | --- | --- | --- | --- | --- |
| **Global Native T1** | 0.82 | 1306.07 | 58.82 | 92.31 | 7.65 | 0.45 |
| **-54.611+0.05*native T1-0.586*PSL** | 0.91 | 1.07 | 82.35 | 100 | - | 0.18 |

+LR: Positive likelihood ratio; -LR: Negative likelihood ratio

**Table 4** Comparison between model diagnosis results and imaging diagnosis results in validation cohort of patients ≥10 years

|  |  | **Diagnostic result** | |  |
| --- | --- | --- | --- | --- |
| **Model** |  | **LGE-** | **LGE+** | **Total** |
| **Global Native T1** | **LGE-** | 4 | 7 | 11 |
|  | **LGE+** | 0 | 1 | 1 |
| **Global Native T1 & PS-Longitudinal** | **LGE-** | 4 | 5 | 9 |
|  | **LGE+** | 0 | 3 | 3 |
| **Total** |  | 4 | 8 | 12 |

**Table 5** Univariable and multivariable logistic regression for the association between LGE and baseline and CMR variables in patients of LVEF <55%

|  | **Univariable OR**  **(95% CI)** | **P**  **value** | **Multivariable OR**  **(95% CI)** | **P**  **value** |
| --- | --- | --- | --- | --- |
| **Age** | 1.81（0.95-3.44） | 0.07§ | 1.57（0.78-3.20） | 0.21 |
| **BMI** | 1.70（0.93-3.12） | 0.08§ | 1.42（0.68-2.99） | 0.35 |
| **HR** | 0.96（0.87-1.08） | 0.51 |  |  |
| **EF** | 0.83（0.63-1.10） | 0.20 |  |  |
| **LV remodeling index** | - | 0.15 | - |  |
| **LVGFI** | 0.85（0.71-1.02） | 0.08§ | 1.02（0.99-1.04） | 0.18 |
| **Global Native T1** | 1.02（1.00-1.04） | 0.12 |  |  |
| **T2** | 1.11（0.67-1.86） | 0.68 |  |  |
| **PS-Radial** | 0.84（0.64-1.10） | 0.20 |  |  |
| **PS-Circumferential** | 1.05（0.62-1.75） | 0.87 |  |  |
| **PS-Longitudinal** | 0.78（0.51-1.20） | 0.26 |  |  |

§p＜0.1

*p＜0.05

** p＜0.01

**Table 6** Univariable and multivariable logistic regression for the association between LGE and baseline and CMR variables in patients of LVEF ≥55%

|  | **Univariable OR**  **(95% CI)** | **P**  **value** | **Multivariable OR**  **(95% CI)** | **P**  **value** |
| --- | --- | --- | --- | --- |
| **Age** | 1.08（0.88-1.34） | 0.43 |  |  |
| **BMI** | 1.06（0.92-1.21） | 0.42 |  |  |
| **HR** | 1.00（0.97-1.03） | 0.88 |  |  |
| **EF** | 0.97（0.89-1.07） | 0.57 |  |  |
| **LV remodeling index** | 0.01（0.00-2.34） | 0.10§ | 0.03（0.00-9.31） | 0.24 |
| **LVGFI** | 1.02（0.95-1.10） | 0.55 |  |  |
| **Global Native T1** | 1.01（1.00-1.02） | 0.06§ | 1.01（1.00-1.02） | 0.11 |
| **T2** | 1.05（0.89-1.24） | 0.56 |  |  |
| **PS-Radial** | 0.92（0.82-1.04） | 0.18 |  |  |
| **PS-Circumferential** | 0.83（0.69-1.00） | 0.05* | 0.86（0.70-1.05） | 0.14 |
| **PS-Longitudinal** | 0.77（0.63-0.95） | 0.02* | 0.81（0.66-1.01） | 0.06 |

§p＜0.1

*p＜0.05

** p＜0.01

**Reviewer #3:**

This is a well-designed study that seeks to solve a real problem in a patient population that is not large.
Although the results are not as encouraging as we would like, the study provides useful information on these cMRI techniques and their clinical significance and is applied to a population of patients that has not been studied in this regard.
The authors also make appropriate reference to the limitations of the study.
For all these reasons, I consider its publication of interest.

**Response:** Thanks for your positive comments.

Now we would like to express our most sincere gratitude for your extraordinary help to polish our work. Thanks again for giving us this chance to make the substantial revisions according to your new comments. We hope the above responses can address your questions properly and please don’t hesitate to contact us if you have any further questions. We will try our best to reply.

Best regards,

Ying-kun Guo, MD
